# Animal models of gut microbiota–male reproductive interactions: methodological assessment, translational bottlenecks, and optimization strategies

**DOI:** 10.3389/fendo.2026.1872670

**Published:** 2026-08-13

**Authors:** Yong Ma, Xuewen Diao, Zulong Wang

**Affiliations:** 1 The First Affiliated Hospital of Henan University of Chinese Medicine Department of Andrology, Zhengzhou, China; 2 The First Clinical Medical School, Henan University of Chinese Medicine, Zhengzhou, China

**Keywords:** animal models, etiological inference, gut–testis axis, idiopathic male infertility, methodological validity, translational modeling

## Abstract

Idiopathic infertility accounts for 30%–40% of male infertility cases, yet its etiology remains largely unexplained. The gut–testis axis hypothesis has recently emerged as a promising direction, with human cohort studies revealing statistical associations between gut dysbiosis and impaired semen quality. However, establishing mechanistic and causal evidence beyond observational associations requires complementary approaches, including animal models. Whether current models adequately capture the chronic, low-grade nature of gut-derived insults that may contribute to idiopathic infertility remains insufficiently examined. Here, we examine the methodological foundations of gut–testis axis animal models. We organize them according to the tier of causal question they address and evaluate their internal, external, and construct validity. Our analysis identifies a recurring pattern in which some existing models, particularly those testing the effects of individual molecular pathways, employ exposure magnitudes, routes, and time scales that may not fully reflect the prolonged, low-intensity disturbances hypothesized in gut-driven reproductive injury. The collective efforts of these model categories have identified several signaling axes that, under defined experimental conditions, can measurably affect the testis. However, the internal and external validity limitations of many of these models warrant caution when interpreting these demonstrations of conditional biological capacity as direct evidence that the same pathways carry etiological weight in human idiopathic infertility. To bridge this gap, we propose a logical framework for evaluating causal evidence that transitions from establishing a pathway’s biological capacity to assessing its etiological contribution. This framework is anchored on three criteria: endogenous signal origin, pathomimetic exposure profile, and attributive exclusivity. We further propose a preliminary reporting framework, termed Minimal Information for Gut–Testis Axis Rodent Studies (MIGTARS), to support more transparent and methodologically informative reporting. These proposals together provide a methodological scaffold for moving the gut–testis field from pathway verification toward translational modeling.

## 1 Introduction

Male infertility has become a global public health issue, with male factors implicated in approximately half of all infertility cases (1). Among these, 30%–40% of infertile men remain without an identifiable cause after comprehensive evaluation and are classified as having idiopathic infertility (2, 3). The management of these patients has long relied on conventional semen analysis, which is largely descriptive, offers limited insight into etiology, and provides few targeted interventions.

In recent years, a link between the gut microbiota and male reproductive health—the “gut–testis axis” hypothesis—has attracted increasing attention. Existing reviews have systematically summarized preclinical evidence that the gut microbiota influences testicular function through pathways including systemic inflammation, oxidative stress, and endocrine disruption (4). In 2025, the concept of the “androbactome” was proposed, referring to the collective gut microbial gene set that may affect androgen synthesis and spermatogenesis (5), reflecting a field-level effort to move from scattered observations toward systematic conceptualization.

More importantly, multi-omics studies from human cohorts have begun to provide preliminary clinical correlates for this hypothesis. The first study integrating the gut and seminal microbiome profiles of men with idiopathic infertility, published in 2021, revealed differential microbial abundances (6). Two recent joint analyses of the seminal microbiome and metabolome further confirmed the co-occurrence of dysbiosis and metabolic disturbances (7, 8). An integrated fecal metagenomic and serum metabolomic analysis of men with asthenozoospermia uncovered a marked depletion of butyrate-producing bacteria and a systemic downregulation of butyrate metabolism pathways (9). Studies on obesity-related metabolic endotoxemia have shown that serum lipopolysaccharide-binding protein (LBP) levels are positively correlated with sperm DNA oxidative damage (10). Collectively, these multi-omics findings from human cohorts demonstrate that both gut-derived metabolites and microbial structural components are statistically associated with semen quality impairment, thereby establishing the clinical relevance of the gut–testis axis.

Although these clinical observations have established preliminary statistical associations between dysbiosis and infertility phenotypes, they remain cross-sectional correlations and cannot determine the direction of causality. In a multicenter seminal microbiome study, Baud et al. likewise noted that the overall composition of the seminal microbiota showed no robust correlation with conventional semen parameters (11). This negative finding highlights a core challenge facing the field: correlative evidence is accumulating, but the causal chain remains undefined.

In parallel with these observational findings, human-based causal inference methods have been increasingly applied to the gut–testis axis. Mendelian randomization studies have begun identifying gut microbial taxa with putatively causal links to male infertility (12). However, the exclusion restriction is difficult to satisfy in this context, as genetic variants influencing microbiota composition may also affect dietary preferences and metabolic profiles through pleiotropic pathways independent of the gut microbiome (13). Longitudinal designs with time-lagged sampling and mediation analyses strengthen temporal inference by establishing that microbial changes precede reproductive outcomes, and can identify delayed effects and quantify indirect pathways (14, 15). Yet these approaches cannot fully exclude unmeasured time-varying confounding, remain sensitive to the choice of time intervals, and struggle to capture complex bidirectional feedback between the gut ecosystem and host physiology (16–18). These approaches can therefore raise the prior probability of causal hypotheses and nominate candidate targets, but fundamental questions of causality remain beyond their reach.

Bridging this causal gap depends critically on experimental animal models. Controlled animal models provide an important component of causal inference by linking microbial perturbations to reproductive phenotypes under experimentally controlled conditions. However, if the animal models that serve as this “evidential foundation” themselves harbor methodological limitations, the clinical translational inferences built upon them must be assessed with caution. This concern is particularly pressing in the field of idiopathic infertility. Molecular pathways of the gut–testis axis have been systematically reviewed (4, 19), but no work has yet examined, from a methodological standpoint, whether the intervention intensity, route of administration, and time scale employed in these models match the prolonged, low-intensity nature of gut-derived insults that may contribute to idiopathic infertility.

This degree of matching determines whether the “mechanisms” verified in these models can be legitimately incorporated into an etiological framework, or whether the evidence merely demonstrates that a pathway is capable of participating in regulation. If a systematic discrepancy exists, directly projecting model-derived conclusions onto the etiological hypotheses of idiopathic infertility may overestimate the strength of evidence and consequently misdirect the prioritization of therapeutic strategies. In contrast to existing reviews centered on molecular mechanisms, this article focuses on a methodological examination of animal models of the gut–testis axis. This line of inquiry runs throughout the entire text and leads to a discussion on proposing a preliminary, domain-specific reporting checklist for gut–testis axis animal studies.

## 2 Literature identification and scope

This study represents a structured methodological narrative review. Rather than aiming to provide quantitative synthesis or estimate pooled effect sizes, the objective was to critically appraise how different animal models of the gut–testis axis address distinct causal questions and how their methodological characteristics influence causal interpretation and translational inference.

Given the heterogeneity of gut–testis axis animal models in intervention strategies, exposure profiles, biological targets, and outcome measurements, quantitative synthesis or direct comparison of effect sizes was not considered appropriate for the objectives of this review.

Relevant literature was identified through searches of PubMed and Web of Science from database inception to June 1, 2026. The search strategy combined terms related to gut microbiota, gut–testis axis, male reproductive function, animal models, microbiota manipulation approaches, microbial metabolites, and genetic dissection strategies. Additional relevant publications were identified through manual screening of reference lists from key articles and reviews.

Literature selection was guided by the methodological objectives of this review. We prioritized original animal studies investigating gut microbiota-related manipulation and reproductive outcomes, including ecosystem-level interventions, pathway-focused interventions, and genetic approaches designed to dissect tissue-specific mechanisms. Studies were selected according to their ability to inform three questions: (1) what causal level does a given model interrogate; (2) what methodological limitations may influence interpretation of the observed phenotype; and (3) to what extent does the experimental design approximate the biological context of human gut–testis axis disorders. Methodological reviews and conceptual papers addressing experimental design, model validity, or translational interpretation were also considered when relevant.

## 3 The preclinical toolbox: a diverse array of models

The clinical associations reviewed above—butyrate-producing bacteria depletion and elevated serum LBP—have motivated a range of animal models. However, the transition from clinically associated factors to experimental causal candidates requires careful consideration. A crucial first step in such an examination is recognizing that not all models address the same tier of causal question. From this perspective, current gut–testis axis models fall into three categories, each answering a distinct question: (1) ecosystem perturbation models—does altering the gut ecosystem disturb testicular function?; (2) pathway probe models—is a specific molecular pathway capable of affecting the testis?; and (3) genetic dissection models—where in the body does the pathway exert its effect? (see **Table 1**).

**Table 1:** Classification framework of gut–testis axis animal models.

Model category	Representative models	Core question addressed
Ecosystem perturbation models	HFD-induced metabolic–microbiota co-perturbation, antibiotic depletion, probiotic supplementation, and FMT	Does altering the gut ecosystem influence testicular function?
Pathway probe models	LPS injection, SCFA supplementation, bile acid intervention	Is a specific molecular pathway capable of influencing testicular function?
Genetic dissection models	Tissue-specific conditional knockout models	At which tissue or cellular site along the gut–testis axis does the pathway exert its effect?

FMT, fecal microbiota transplantation; HFD, high-fat diet; LPS, lipopolysaccharide; SCFA, short-chain fatty acid.

### 3.1 Ecosystem perturbation models: altering the gut ecosystem to query reproductive effects

Ecosystem perturbation models operate at the level of the entire gut microbial ecosystem or the host–microbiota metabolic interface. Rather than isolating single molecules or pathways, they increase, deplete, or reconfigure the gut microbiota as a whole, asking whether such alterations disturb testicular function.

High-fat diet (HFD)–induced metabolic–microbiota co-perturbation. Feeding mice a HFD (60% kcal from fat) for 16–20 weeks has become the mainstream paradigm for studying metabolically driven disruption of the gut–testis axis. The model reliably produces intestinal barrier damage (elevated zonulin, decreased occludin), low-grade elevation of circulating lipopolysaccharide (LPS), increased blood–testis barrier (BTB) permeability, reduced sperm counts, and upregulation of testicular pro-inflammatory cytokines such as TNF-α and IL-1β (20, 21). A recent study further showed that the reproductive toxicity of paternal HFD-induced metabolic stress can extend beyond spermatogenesis, markedly delaying preimplantation developmental timing and perturbing the expression of implantation-related genes in the maternal uterus (22).

Administering an antibiotic cocktail composed of ampicillin, vancomycin, neomycin, and metronidazole (AVNM) in drinking water for 2–6 weeks is the standard regimen for depleting the gut microbiota. Some studies have documented seminiferous epithelial damage in mice under this regimen (23). Conceptually, this model probes the opposite direction of the ecosystem-level question: if HFD asks whether metabolic–microbiota dysbiosis damages the testis, antibiotic depletion asks whether removing the microbiota also damages the testis.

Microbial supplementation models, represented by oral gavage of specific probiotic strains (e.g., Lactiplantibacillus plantarum) or fecal microbiota transplantation (FMT) from healthy donors, have consistently reported improved sperm quality and preserved testicular structure under various stress conditions (24, 25). A recent systematic review of randomized clinical trials corroborated these findings, demonstrating that probiotic intervention significantly improves sperm motility and reduces oxidative stress in men diagnosed with idiopathic infertility (26). FMT, in particular, can simulate causal transmission of the microbiota, offering a unique tool for establishing whether microbiota alterations are sufficient to drive reproductive phenotypes; notably, FMT has been shown to restore BTB integrity disrupted by antibiotic-induced gut dysbiosis in mice (27).

### 3.2 Pathway probe models: testing whether specific molecules can affect the testis

In contrast to ecosystem perturbation models, which manipulate the entire gut ecology, pathway probe models bypass the microbiota and directly administer a single molecule to test whether a specific signaling axis is capable of affecting testicular function. These models bypass the microbiota itself and directly administer candidate metabolites to verify the regulatory effects of specific signaling axes on the testis; they represent one of the most active model groups at present. Intraperitoneal injection of LPS(typically 0.5–5.0 mg/kg as a single bolus) induces acute BTB disruption within 24 hours and is the most widely used endotoxin–testicular injury model (28). Beyond these, multiple other metabolite-based models are in use. Supplementation with short-chain fatty acids (SCFAs), most commonly oral gavage of sodium butyrate, has been reported to improve sperm motility and restore BTB integrity in mice with HFD-induced reproductive damage (21). Bile acid intervention, for instance, dietary supplementation with cholic acid, has been shown to impair testicular function and fertility in mice (29).

### 3.3 Genetic dissection models: pinpointing the site of action

Ecological perturbation models address the question of whether altering the gut ecosystem can disturb testicular function. Pathway probe models address the question of whether a specific molecule is capable of affecting the testis. Together, however, they leave a critical question unanswered: where exactly along the gut-to-testis signaling axis does the key event occur? When a microbial metabolite is detected in serum or seminal plasma, it may act via two fundamentally different routes. It may initiate a signaling cascade at the intestinal epithelium, which is then relayed indirectly to the testis by secondary mediators or immune cells. Alternatively, it may enter the systemic circulation directly and act locally within the testicular microenvironment on Sertoli or Leydig cells. The first two model categories cannot distinguish between these possibilities.

Genetic dissection models are designed precisely to spatially decouple a gut-derived distant effect from a local, direct testicular effect. The core strategy is to block the same signaling pathway at the intestinal end and at the testicular end, and then determine at which end the reproductive phenotype is abolished. This strategy is operationalized using the tissue-specific Cre-loxP system (30). Conceptually, the system works like a pair of molecular scissors: the Cre recombinase is engineered to cut DNA only at specific sites (loxP sites), and its expression is restricted to a chosen tissue by a tissue-specific promoter (31). Thus, a gene can be deleted only in intestinal epithelial cells (using, e.g., a Vil1-Cre driver) or only in testicular somatic cells (using, e.g., an Amh-Cre driver for Sertoli cells or Cyp17-iCre for Leydig cells (31), while remaining intact elsewhere in the body. By comparing the reproductive phenotype when the same pathway is disrupted in the gut versus in the testis, one can determine whether the critical signal originates from the intestinal lining or acts directly within the testicular microenvironment. This spatial decoupling is the unique contribution of genetic dissection models, a capability that neither ecological perturbation nor pathway-probe models possess.

Taking the LPS-TLR4 pathway as an example, Luo et al. generated Sertoli cell-specific Myd88 conditional knockout mice. Upon LPS stimulation, these conditional knockout mice showed marked attenuation of testicular inflammation, BTB disruption, and spermatogenic damage, thereby identifying Sertoli cells as the key effector cells of this pathway within the testis (32). A similar genetic decoupling strategy can be extended to other gut–testis axis signaling pathways, including SCFA–G protein-coupled receptor, bile acid–farnesoid X receptor/G protein-coupled bile acid receptor 1, and tryptophan–aryl hydrocarbon receptor pathways, whose receptors are co-expressed in both the intestinal epithelium and testicular somatic cells (33–36).

Taken together, these three model categories form a progressive loop of causal inquiry: ecological perturbation models ask whether a link exists; pathway probe models ask which molecule is capable of mediating it; and genetic dissection models ask where the signal acts.

## 4 Methodological constraints and translational bottlenecks

### 4.1 Erosion of internal validity: confounding, toxicity, uncontrolled FMT, and genetic model artifacts

As discussed above, the three categories of gut–testis axis animal models address distinct tiers of causal question. However, each category faces its own sources of confounding within its experimental framework, undermining the reliability of causal inference from the intended manipulation to the observed reproductive phenotype. In ecological perturbation models, the dominant threats are coprophagy-mediated microbial exchange, metabolic comorbidities embedded in diet-induced obesity protocols, and the direct pharmacological toxicity of gut-depleting agents. In genetic dissection models, the corresponding concerns center on developmental compensation, off-target recombination, and induction-related artifacts. We examine these in turn.

#### 4.1.1 Overlooked coprophagy and cage effects

Coprophagy in mice essentially constitutes an uncontrolled form of “within-group fecal microbiota transplantation”: co-housed animals continuously exchange microbes through stool consumption, homogenizing the microbiota across groups and thereby potentially obscuring or systematically biasing the estimated effect size (37, 38). Even when animals are singly housed, cage effects can still become a source of microbiota variation stronger than the experimental intervention if randomization is not implemented at the cage level. Many studies fail to report housing arrangements, the number of animals per cage, or statistical correction for cage effects; only a limited number of microbiome studies have explicitly described cage-level randomization schemes (39). This oversight poses a direct threat to the causal validity of fecal microbiota transplantation experiments: if donors and controls are co-housed, the “signature” of the donor microbiota ceases to exist.

#### 4.1.2 Metabolic confounding in high-fat diet models

Although the HFD model reliably induces testicular damage, interpreting the phenotype as a unidirectional causal chain of “HFD → gut dysbiosis → testicular injury” lacks the necessary attributional constraint. Multiple microbiota-independent pathways can independently cause spermatogenic impairment. Upregulated aromatase activity in adipose tissue promotes the conversion of testosterone to estradiol, directly suppressing the hypothalamic–pituitary–gonadal axis (40). Different dietary fat sources—lard versus soybean oil, for instance—can exert independent effects on the testis through distinct lipid metabolic routes (41). The substantial dilution of dietary fiber in high-fat chow is itself an independent variable that reshapes microbial community structure and reduces SCFA production, yet its effect is often indiscriminately subsumed under the “HFD” label (42). Moreover, obesity, insulin resistance, and gut dysbiosis are reciprocally causal, making adequate decoupling through statistical adjustment difficult (6).

To improve causal interpretation, future studies should provide detailed descriptions of dietary composition, including fat source, macronutrient distribution, caloric density, and fiber content, as dietary formulation itself can influence microbial and metabolic phenotypes independently of obesity development (43). Given the heterogeneity of HFD models, careful consideration of fat source and dietary composition is essential when interpreting metabolic and reproductive outcomes (44, 45). When the experimental question specifically concerns microbiota-mediated mechanisms, approaches such as pair-fed controls, longitudinal metabolic phenotyping, and microbiota-independent validation may further help distinguish microbial effects from broader metabolic consequences of HFD exposure.

#### 4.1.3 Direct toxicity confounding in antibiotic-based models

A major interpretative challenge of broad-spectrum antibiotic cocktail models is the potential overlap between microbiota depletion effects and direct host responses to antibiotic exposure. Taking the AVNM regimen as an example, antibiotics with different pharmacological properties may influence host physiology beyond their effects on microbial communities. Although the extent of systemic exposure varies depending on antibiotic type, dose, and administration route, several antibiotic classes have been associated with reproductive toxicity, including alterations in steroidogenic pathways, oxidative stress regulation, and hypothalamic–pituitary–gonadal axis function (46–48). Therefore, reproductive phenotypes observed after antibiotic depletion may reflect microbiota-dependent mechanisms, direct pharmacological effects, or a combination of both.

To strengthen causal interpretation, future studies using antibiotic-based depletion models should verify the extent and stability of microbiota depletion, as antibiotic composition, dosage, and administration route can substantially influence depletion efficiency (49, 50). Incorporating recovery periods after antibiotic exposure and complementary approaches such as microbiota restoration or targeted microbial metabolite interventions may further help distinguish microbiota-dependent effects from direct pharmacological consequences (51).

#### 4.1.4 Overlooked confounders in genetic dissection models

Genetic dissection models, despite providing high spatial and molecular resolution, are not immune to the distinction between demonstrating biological capacity and establishing etiological relevance. Their methodological constraints therefore require careful consideration.

First, constitutive knockout from embryogenesis may induce developmental compensation, thereby obscuring the contribution of a pathway to adult physiological function. A representative finding in the reproductive system illustrates this possibility: Cyp17a1-Cre-mediated, Leydig cell-specific deletion of CREBZF resulted in largely preserved *in vivo* fertility and serum testosterone levels, whereas primary Leydig cells isolated from these animals displayed impaired testosterone synthesis. This discrepancy suggests that compensatory mechanisms may operate at the organismal level, potentially including enhanced adrenal steroidogenic activity (52). Therefore, inducible models or complementary approaches comparing developmental and adult-onset deletion may be valuable when investigating adult reproductive mechanisms.

Second, the assumption of tissue specificity in Cre-loxP systems is frequently challenged by unexpected Cre activity. The Amhr2-iCre line, widely used in reproductive research, has been reported to induce genetic modification beyond the intended tissue lineage, including activity during early embryonic stages (53). Similarly, the 8kb Dmp1-Cre line showed unexpected activity in intestinal mesenchyme, and deletion of Tsc1 using this model resulted in colonic phenotypes that could potentially be misinterpreted as tissue-specific effects (54). These observations highlight the importance of validating recombination patterns experimentally and including appropriate Cre-expressing control groups when interpreting conditional knockout phenotypes.

Third, inducible systems, while designed to minimize developmental compensation, introduce additional variables. These include potential cellular effects associated with CreERT2 activation itself (55) and endocrine effects induced by tamoxifen administration (56). Accordingly, tamoxifen-treated Cre-negative controls and appropriate temporal validation are important for distinguishing gene-specific effects from induction-related artifacts.

Collectively, these considerations indicate that loss-of-function phenotypes in genetic dissection models should be interpreted within the context of model validation rather than as direct evidence of pathway-specific necessity alone. Careful assessment of developmental compensation, Cre specificity, and induction-related effects, together with complementary validation strategies such as rescue experiments or independent genetic approaches, can strengthen causal inference from these models (57).

### 4.2 Limitations in external validity: mismatches between experimental models and human disease contexts

#### 4.2.1 Exposure mismatch: acute experimental perturbation versus chronic disease trajectory

Even when internal validity is adequately addressed, the translational relevance of animal models depends on whether the experimental context corresponds to key characteristics of the human disease process. For gut–testis axis disorders associated with idiopathic infertility, a major challenge is the mismatch between experimentally imposed exposures and the chronic, low-intensity disturbances hypothesized to occur in humans. The observation window of acute pathway-probe models often represents a substantial departure from this clinical context. For example, acute LPS models typically induce testicular inflammatory responses within 24–72 hours, whereas antibiotic-based depletion protocols generally last 2–6 weeks. These exposure windows are comparable to or shorter than a single complete spermatogenic cycle in mice (~35 days), and therefore may preferentially capture acute or subacute responses rather than progressive alterations associated with chronic disease development (58).

**Figure 1:**
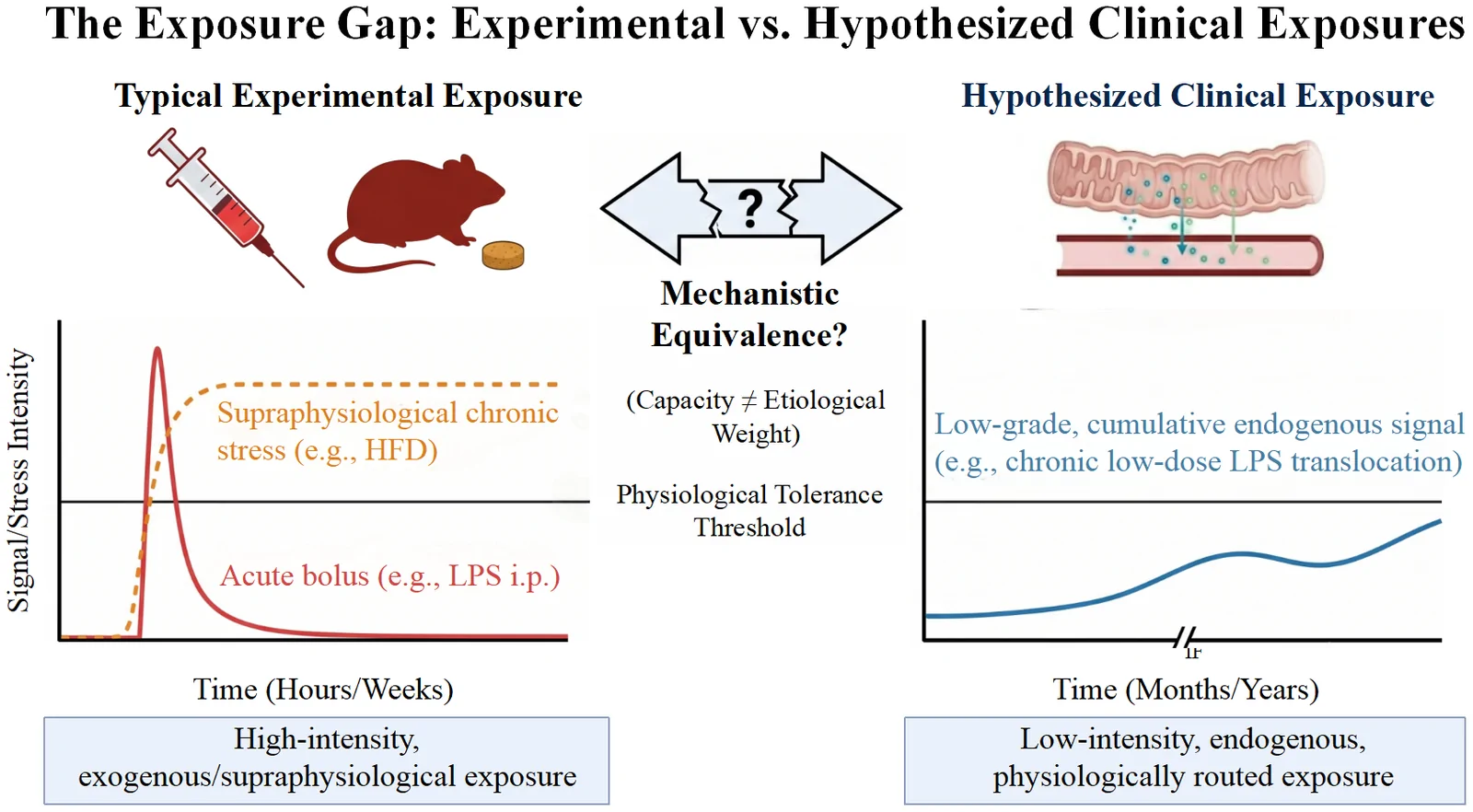
The exposure gap: experimental vs. hypothesized clinical exposures.

Although the HFD model (16–24 weeks) and SCFA supplementation protocols (≥8 weeks) encompass multiple cycles in duration, most studies still rely on single terminal sampling. The dynamic evolution of damage—whether progressive or plateauing, whether compensatory adaptation occurs, and whether recovery is possible after withdrawal of intervention—remains poorly characterized. This limitation restricts the ability to determine whether a given pathway represents a sustained driver of reproductive dysfunction or merely a transient modulator under specific experimental conditions. This disparity is illustrated in **Figure 1**.

#### 4.2.2 Cross-species and disease-context differences

Beyond exposure duration and disease trajectory, cross-species biological differences represent another important limitation for translational interpretation (59). Rodent models provide indispensable experimental platforms for dissecting gut–testis interactions; however, the biological context in which these interactions occur is not identical between rodents and humans.

First, substantial differences exist between rodent and human gut microbial ecosystems (60, 61). Laboratory rodents are maintained under highly controlled conditions and are influenced by factors such as standardized diets, housing environment, and coprophagy behavior, whereas human microbiota are shaped by diverse dietary patterns, environmental exposures, and lifestyle factors (62). These ecological differences may influence microbial community structure, metabolite production, and the systemic availability of gut-derived signals.

Second, reproductive physiology differs between rodents and humans (58, 63). Differences in spermatogenic duration, sperm maturation kinetics, reproductive tract anatomy, and accessory gland biology may influence how microbial-derived signals affect reproductive outcomes. Therefore, reproductive phenotypes observed in rodents should not be interpreted as direct equivalents of human infertility phenotypes without considering these physiological differences.

Finally, the clinical context of idiopathic infertility itself presents a translational challenge. Human idiopathic infertility is a heterogeneous and multifactorial condition influenced by genetic susceptibility, lifestyle factors, environmental exposures, and age-related changes (64). In contrast, most rodent models investigate a single controlled perturbation on a relatively uniform genetic background. Thus, these models are best interpreted as tools for dissecting specific biological components of reproductive dysfunction rather than complete representations of human infertility.

### 4.3 Construct validity concerns: distinguishing biological capacity from etiological contribution

Animal models remain indispensable for dissecting gut–testis interactions; however, their conclusions need to be interpreted according to the causal level that each model is designed to address. Construct validity asks whether an experimental model actually measures or simulates the concept it claims to investigate.

According to the classification framework presented in Section 3, the construct of pathway probe models is clear: they verify whether a specific molecular pathway is capable of influencing testicular function. Yet even a methodologically sound pathway probe experiment may be subject to interpretative overextension if its findings are equated with evidence that the pathway materially contributes to disease pathogenesis. The distinction between these two propositions reflects different levels of causal interpretation. The former establishes biological capacity under defined experimental conditions; the latter requires assessment of causal weight within the context of disease development.

This distinction has not always been explicitly considered in interpretations of gut–testis axis studies. For instance, one study disrupted the gut microbiota using broad-spectrum antibiotics or HFD-induced metabolic disturbance, followed by a single acute high-dose intraperitoneal LPS injection to induce testicular injury, and interpreted the findings as indicating that “the gut microbiota participates in the pathogenesis of orchitis” (27). Other studies have drawn strong causal links between HFD-induced microbial dysbiosis and testicular ferroptosis while the contribution of accompanying metabolic changes remained difficult to separate from microbiota-mediated effects (65).

Genetic dissection models, despite their spatial precision, also require careful interpretation within this framework. By demonstrating that testicular damage is abolished when a signaling pathway is blocked specifically in Sertoli cells, such experiments rigorously establish where the signal acts and whether the targeted cell type is necessary for the observed injury. However, in an etiological framework, the question is not whether a pathway can be rendered non-functional, but whether its endogenous, gut-originated activation profile in the natural disease course is sufficient to drive pathogenesis. Constitutive conditional knockout designs may introduce developmental adaptations and do not necessarily recapitulate the gradual, low-grade signal dysregulation hypothesized in idiopathic infertility. The inference that “because a pathway is necessary for experimental injury, it must therefore contribute etiological weight to the natural disease” represents a similar interpretative challenge across different model categories.

What such experimental designs can rigorously demonstrate is that, under experimentally imposed conditions such as acute endotoxin exposure or severe metabolic perturbation, the state of the microbiota can modulate the organism’s response to that insult. However, interpreting such findings directly as evidence of etiological relevance may exceed what the experimental design can independently establish. The conditions these experiments simulate—a systemic septic complication or a severe obesity model—may differ substantially from the slowly progressive, non-infectious clinical context of idiopathic infertility. The former indicates that microbiota status can modulate susceptibility under experimentally induced stress conditions, whereas the latter asks whether the microbiota can act as an endogenous chronic driver that independently propels the disease course. These represent different levels of causal inference rather than equivalent etiological scenarios.

It is important to state clearly that the criticism here is directed at the potential mismatch between experimental conclusions and the level of causal inference they support, not at the value of the experiments themselves. The studies cited above have made valuable contributions by demonstrating the biological potential of specific pathways or microbial communities to regulate reproductive function. In this context, the term “pathogenesis” generally implies mechanisms involved in disease initiation and progression, rather than factors that merely modulate the severity of an established condition. When findings from pathway probe models are framed in etiological language, “biological capability” is implicitly elevated to “etiological weight,” which in turn may misdirect future research investment—prompting the field to repeatedly verify which pathways are capable, while delaying the question of how much of that capability is actually realized in the real disease state.

After completing these three levels of validity scrutiny, a balanced acknowledgement is in order. Taken together, these validity analyses do not imply that the gut–testis axis hypothesis is unfounded. Rather, they reveal a systematic mismatch between what current models can rigorously demonstrate and the broader conclusions that may be inferred from them. Pathway probe and ecological perturbation models have shown that, under conditions of high-intensity, often non-physiological exposure, certain molecular axes are capable of affecting the testis. Whether—and to what extent—these same pathways are actually engaged by the low-grade, chronic gut-derived signals hypothesized in idiopathic infertility remains an open question. Closing this gap will likely require re-validating key pathways under exposure profiles that more closely mimic the chronicity and intensity of the clinical condition, together with additional evidence regarding endogenous signal generation and disease context.

## 5 Discussion and perspectives

### 5.1 From “capacity” to “weight”: a logical framework for evaluating causal evidence

**Figure 2:**
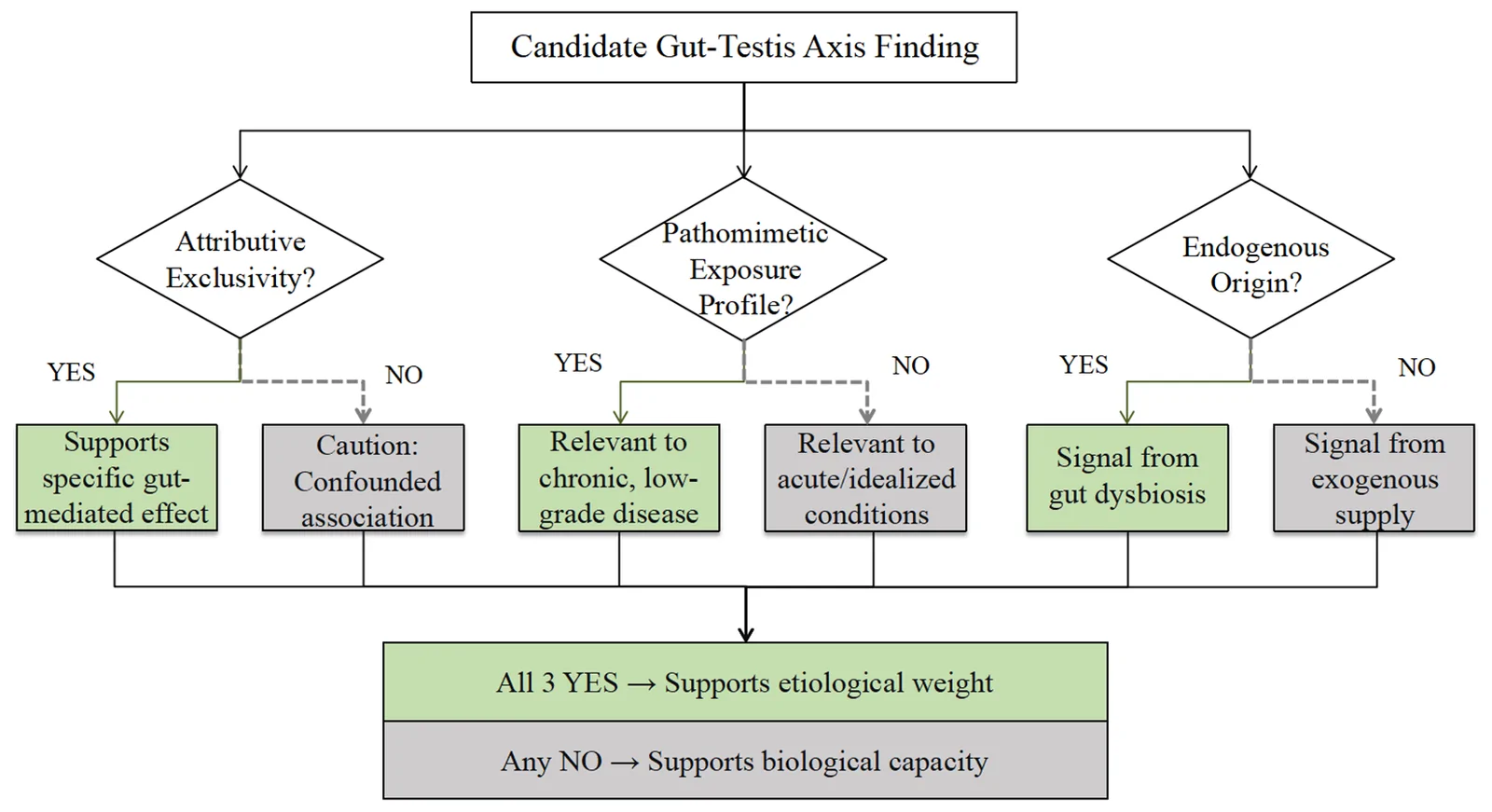
Logical evaluation framework for assessing causal evidence in gut-testis axis studies.

When designing a gut–testis axis study or evaluating its conclusions, researchers therefore need to consider: exactly which step along the causal chain from capacity verification to weight establishment has this work reached? To this end, we propose a logical evaluation framework comprising three parallel criteria (**Figure 2**). These criteria are not intended as rigid pass/fail thresholds, but rather as a spectrum of evidence strength to help researchers reflect on the interpretative boundaries of their findings.

Attributive exclusivity (internal validity). When a model induces changes in the gut microbiota alongside systemic metabolic disturbances, unintended pharmacological toxicity, or other systemic stresses, the observed reproductive damage may have multiple explanations. Ideally, a model should reliably decouple the effect of the gut microbiota from these confounders. When this criterion is clearly met, the evidence for a specific gut-mediated effect is strengthened. When it is not fully met, the findings should be interpreted as showing a statistical association that may be confounded, and causal inference requires caution.

Pathomimetic exposure profile (external validity). If gut-derived factors contribute to idiopathic infertility, their mode of action is likely to involve chronic, low-intensity insults rather than acute, high-magnitude challenges. Therefore, a key consideration is whether the exposure imposed by a model—in magnitude, duration, and route—reasonably approximates the physiological range of gut dysbiosis. When a model meets this criterion, its findings are more likely to be relevant to the natural disease course. When it does not, the model demonstrates that gut microbiota alterations have biological capacity under idealized or acute conditions, but their etiological role in chronic, low-grade human disease remains to be established.

Endogenous origin of the signal (construct validity). The candidate pathogenic signal should ideally originate from disordered host–microbiota interactions within the gut ecosystem, rather than being forcibly introduced by exogenous administration. When a model demonstrates that a signal is generated endogenously from gut dysbiosis, it strengthens the case for etiological relevance. When the signal is delivered exogenously, the findings confirm that gut-derived factors can affect the testis under certain conditions, but they do not directly support the hypothesis that the same factors are endogenously activated in human idiopathic infertility.

Synthesizing the criteria. Confidence that a gut-mediated effect carries etiological weight is greatest when all three criteria are adequately supported. When one or more criteria remain insufficiently addressed, conclusions should be calibrated to the available evidence, which may support biological capacity more strongly than etiological contribution. This distinction is not a value judgment on the quality of the study, but rather a necessary clarification of what conclusions can legitimately be drawn from its findings. **Figure 2** provides a visual summary of this logical framework.

### 5.2 Establishing the minimal information for gut–testis axis rodent studies

Progress at the model level must be built on the assurance of research quality. The internal validity defects identified in Section 4—uncontrolled microbial exchange driven by coprophagy, direct reproductive toxicity of antibiotics, supraphysiological dosing in metabolite interventions, missing donor information in FMT studies, and paradigm confusion arising from unclear model positioning—are not a collection of unrelated oversights. Rather, they are the systemic manifestation of a field-level gap: the absence of a set of minimum reporting standards that can safeguard experimental reproducibility and translational relevance. Most of these problems cannot be eliminated through technical optimization within individual studies, because they concern the foundational infrastructural conditions of experimental transparency and cross-study comparability.

We therefore propose the Minimal Information for Gut–Testis Axis Rodent Studies (MIGTARS) as a preliminary, domain-specific reporting checklist for gut–testis axis animal research. MIGTARS is not intended to replace established general frameworks such as Animal Research: Reporting of *In Vivo* Experiments (ARRIVE), but to complement them by highlighting methodological details that are particularly important for interpreting microbiota–reproduction experiments. Drawing on the validity concerns identified in Section 4, the checklist is organized around model positioning, ecosystem-level interventions, pathway-probe studies, and genetic dissection models, with concrete reporting items linked to the corresponding threats to internal, external, or construct validity (**Table 2**). These items address issues such as cage-level clustering and microbial exchange, antibiotic exposure and potential direct toxicity, dietary composition, FMT donor characterization, dose and administration-route relevance, and validation of tissue-specific genetic models. MIGTARS does not attempt to reproduce all general animal-study reporting requirements already addressed by ARRIVE; rather, it focuses on domain-specific information that may materially influence causal interpretation and cross-study comparison in gut–testis axis research. The proposed items should be regarded as exploratory recommendations intended to facilitate transparent reporting and critical appraisal, rather than as mandatory standards. Their further refinement will require broader discussion, empirical evaluation, and consensus within the field.

**Table 2:** MIGTARS: A proposed checklist for reporting gut–testis axis rodent studies.

Domain	Checklist item	Recommended reporting requirement	Primary validity domain
General	Model positioning	Define the causal question addressed and specify whether the model evaluates ecosystem perturbation, pathway capacity, or tissue-specific mechanism.	Construct
Ecosystem models	Cage-effect control	Report housing conditions, cage number, cage-level randomization, and statistical handling of cage effects.	Internal
Ecosystem models	Antibiotic exposure and toxicity control	Report antibiotic composition, duration, absorption characteristics, and approaches used to address direct reproductive toxicity.	Internal
Ecosystem models	Diet composition disclosure	Report fat source, fat content, fiber content/source, and matched control diet composition.	Internal
Ecosystem models	FMT donor characterization and separation	Report donor selection, microbiota characterization, housing separation, and procedures minimizing microbial exchange.	Internal
Pathway probe models	Dose justification and physiological relevance	Provide dose rationale and compare experimental exposure with physiological/pathological ranges.	External
Pathway probe models	Administration route relevance	Describe whether the route reflects endogenous exposure and discuss pharmacokinetic limitations.	External
Genetic models	Genetic model validation	Report Cre specificity, recombination efficiency, developmental compensation, and inducer-related effects.	Internal & Construct

Several items in **Table 2** warrant further operational clarification to facilitate their application and interpretation. For cage-effect control, we recommend that cage identity be modeled as a random effect in mixed-effects models (39, 57); when within-cage sample sizes are small, cage-level summary statistics may provide a pragmatic alternative when mixed-effects modeling is not feasible (37). The essential principle is that the clustering structure of the data should be transparently reported and appropriately accounted for in the analysis.

Antibiotics commonly regarded as having limited oral bioavailability include vancomycin, neomycin, bacitracin, and polymyxin B. That said, even non-absorbable antibiotics may generate intestinal luminal alterations that indirectly affect host physiology. A more broadly applicable reporting consideration is therefore that studies should explicitly report the systemic absorption characteristics of the antibiotics used and discuss residual confounding from direct reproductive toxicity.

Regarding HFD composition disclosure, we refrain from prescribing a universal fiber threshold, as the appropriate range depends on strain, facility, and metabolic context. Instead, we recommend that studies report the fiber content and source type (soluble vs. insoluble) of both the experimental and control diets, and explicitly state whether these are matched. A mismatch should be acknowledged as a potential confounder in the discussion. The source and proportion of dietary fat should likewise be reported, because different lipid compositions may exert microbiota-independent metabolic and reproductive effects.

Report the administered dose and route of administration. Where pharmacokinetic or tissue-concentration data are available, compare the estimated exposure profile with relevant physiological or pathological concentration ranges and report the fold difference where appropriate. Any potential implications of supraphysiological exposure for mechanistic interpretation and translational relevance should be explicitly discussed.

For the genetic model validation item, we do not propose *de novo* methodological standards. We instead recommend that authors consult established safety and validation frameworks, such as that proposed by Horvath et al. Although developed for metabolic research, that framework is generalizable: it covers Cre driver specificity, developmental compensation, and inducer-related confounds—all of which are equally relevant to gut–testis axis studies employing conditional knockouts (57). We recommend consulting this framework when reporting the item.

As proof-of-principle, existing studies already demonstrate the feasibility of the proposed reporting items. For instance, individually ventilated cages with documented cage-level randomization have been adopted in microbiome research (66), and Argaw-Denboba et al. employed a non-absorbable antibiotic cocktail (neomycin, bacitracin, pimaricin) with mass-spectrometric confirmation that systemic exposure was not detectable under the experimental conditions (67). These examples demonstrate that the proposed checklist items are feasible to implement under real-world experimental conditions.

Collectively, these reporting items are intended to make the assumptions, exposure characteristics, and inferential boundaries of gut–testis axis models more transparent. Their use may improve cross-study comparison and help distinguish evidence of biological capacity from evidence supporting etiological contribution. However, reporting improvements alone cannot resolve limitations inherent in model design. Progress toward translationally informative inference will also require experimental models that more closely approximate clinically relevant exposure magnitude, route, duration, and endogenous signal generation.

### 5.3 Limitations of this review

This review has several limitations. First, as a structured methodological narrative review, it was designed to evaluate the causal interpretability and methodological validity of existing gut–testis axis animal models rather than to provide quantitative synthesis of effect sizes. Therefore, no meta-analysis was performed, and the relative strength of evidence across different model categories could not be statistically compared.

Second, although literature identification was guided by predefined methodological objectives, the selection and interpretation of studies inevitably involved expert judgment regarding model relevance, causal inference, and translational value. Therefore, some degree of interpretive subjectivity cannot be completely excluded.

Third, the proposed MIGTARS framework represents an initial conceptual and reporting framework rather than a formally validated guideline. Its applicability, feasibility, and impact on reporting quality require further evaluation through broader adoption and future consensus-building processes. Finally, as the gut–testis axis field continues to evolve, emerging models and technologies may further refine the assessment criteria proposed here.

## 6 Conclusion

In conclusion, current gut–testis axis animal models provide valuable evidence that microbial alterations and related signaling pathways can influence testicular function under defined experimental conditions; however, their interpretation should remain aligned with the causal question and biological context that each model is designed to address. Future progress in this field will depend on improving translational model relevance and establishing transparent reporting standards, such as the proposed MIGTARS framework, to strengthen causal inference and methodological reproducibility.
